# An excitatory amacrine cell detects object motion and provides feature-selective input to ganglion cells in the mouse retina

**DOI:** 10.7554/eLife.08025

**Published:** 2015-05-19

**Authors:** Tahnbee Kim, Florentina Soto, Daniel Kerschensteiner

**Affiliations:** 1 Department of Ophthalmology and Visual Sciences, Washington University School of Medicine, Saint Louis, United States; 2 Graduate Program in Neuroscience, Washington University School of Medicine, Saint Louis, United States; 3 Department of Anatomy and Neurobiology, Washington University School of Medicine, Saint Louis, United States; 4 Hope Center for Neurological Disorders, Washington University School of Medicine, Saint Louis, United States; Max Planck Institute of Neurobiology, Germany

**Keywords:** retinal circuitry, amacrine cell, feature detection, VGluT3, mouse

## Abstract

Retinal circuits detect salient features of the visual world and report them to the brain through spike trains of retinal ganglion cells. The most abundant ganglion cell type in mice, the so-called W3 ganglion cell, selectively responds to movements of small objects. Where and how object motion sensitivity arises in the retina is incompletely understood. In this study, we use 2-photon-guided patch-clamp recordings to characterize responses of vesicular glutamate transporter 3 (VGluT3)-expressing amacrine cells (ACs) to a broad set of visual stimuli. We find that these ACs are object motion sensitive and analyze the synaptic mechanisms underlying this computation. Anatomical circuit reconstructions suggest that VGluT3-expressing ACs form glutamatergic synapses with W3 ganglion cells, and targeted recordings show that the tuning of W3 ganglion cells' excitatory input matches that of VGluT3-expressing ACs' responses. Synaptic excitation of W3 ganglion cells is diminished, and responses to object motion are suppressed in mice lacking VGluT3. Object motion, thus, is first detected by VGluT3-expressing ACs, which provide feature-selective excitatory input to W3 ganglion cells.

**DOI:** http://dx.doi.org/10.7554/eLife.08025.001

## eLife digest

Animals can use their eyes to detect moving objects, which helps them to avoid predators and other threats, and to spot potential prey or allies. Visual information from the eyes is sent to the brain, which processes the information to form a coherent picture of how the objects are moving. This processing has to be able to account for movements of the head, eyes, and body—which can cause the image of an object on the retina within the eye to move even if the object itself remains stationary.

Within the retina, light is converted into electrical signals by cells called rods and cones. A layer of cells called bipolar cells relay these signals to the ‘ganglion’ cells, which in turn pass them on to the brain. In mice, a type of ganglion cell called the W3 ganglion cell has been shown to respond selectively to small moving objects, but exactly how these cells acquire their motion sensitivity remained unclear.

Kim et al. now reveal that cells called amacrine cells, which regulate the transfer of signals from the bipolar cells to ganglion cells, supply the information needed for motion detection. The mouse eye contains up to 50 different types of amacrine cells. One of these—called the VG3-amacrine cell—increases its activity whenever an object moves relative to its background, but decreases its activity whenever the object and background move together. The overall effect is that the cells respond selectively to the presence of small moving objects.

Most amacrine cells regulate the transfer of signals within the retina by inhibiting the activity of ganglion cells. But, Kim et al. show that VG3-amacrine cells release a molecule called glutamate to activate W3 ganglion cells when a moving object is detected. These unusual and specialized cells are, thus, an essential component of a circuit in the nervous system that supports motion detection. It is possible that some other types of amacrine cells may also play specialized roles in the detection of other features in the visual world.

**DOI:** http://dx.doi.org/10.7554/eLife.08025.002

## Introduction

A diverse array of circuits in the retina processes signals from photoreceptors and parses information into spike trains of 20–30 types of retinal ganglion cells (RGCs), each encoding distinct aspects of the visual scene (Masland, 2012). The most abundant RGC type in the mouse retina (W3-RGC) was recently shown to respond selectively to movements of small—in terms of size on the retina—objects (Zhang et al., 2012). Detecting object motion is a challenging task as head, body, and eye movements frequently shift the retinal image (Martinez-Conde et al., 2004; Sakatani and Isa, 2007). To distinguish movements of objects and the background, object motion sensitive (OMS) RGCs respond to differences in the timing of texture movements in their receptive field center and surround (Olveczky et al., 2003; Zhang et al., 2012). W3-RGCs share key properties with OMS RGCs in rabbit and salamander, but, due to stronger surround suppression, do not respond at the border of larger objects (Zhang et al., 2012). This feature is reminiscent of local-edge-detector RGCs described in several species (Levick, 1967; Zeck et al., 2005; Roska et al., 2006). W3-RGCs, thus, appear to be in the intersection of OMS and local-edge-detector RGCs. Although postsynaptic inhibition and spike thresholds sharpen the tuning of W3-RGCs, similar to other OMS and local-edge-detector RGCs, key response properties appear to be inherited from their excitatory input (van Wyk et al., 2006; Baccus et al., 2008; Russell and Werblin, 2010; Zhang et al., 2012). This suggests that feature selectivity arises presynaptic to W3-RGCs. Where and how object motion is first detected remains to be determined.

Typically, RGCs receive excitatory input from bipolar cells (Euler et al., 2014) and inhibitory input from amacrine cells (ACs). ACs are the most diverse class of neurons in the retina, encompassing 30–50 cell types (MacNeil and Masland, 1998; Helmstaedter et al., 2013) that serve task-specific functions in vision (Dacheux and Raviola, 1986; Yoshida et al., 2001; Euler et al., 2002; Munch et al., 2009; Grimes et al., 2010; Chen and Li, 2012). Although most ACs release γ-Aminobutyric acid (GABA) or glycine, a wide range of neurotransmitters and neuromodulators can be found in different cell types including one expressing the vesicular glutamate transporter 3 (VGluT3) (gene: *Slc17a8*, protein: VGluT3, AC: VG3) (Fyk-Kolodziej et al., 2004; Haverkamp and Wassle, 2004; Johnson et al., 2004). VG3-ACs are conserved from rodents to primates. Recent studies found that VG3-ACs respond to light increments (ON) and decrements (OFF) (Grimes et al., 2011), show strong surround suppression, and following optogenetic or electrical stimulation can release glutamate (Lee et al., 2014). However, what specific features of the visual world VG3-ACs detect and how, as well as their interactions with RGCs during sensory processing remain unknown.

Here, we generate and obtain transgenic mouse lines to genetically label VG3-ACs and target them under 2-photon guidance for whole-cell patch-clamp recordings in retinal flat mount preparations. We find that VG3-ACs, like W3-RGCs, combine properties of OMS and local-edge-detector neurons and selectively detect movements of small objects. Using biolistics, we show that many excitatory synapses on W3-RGCs are apposed by boutons of VG3-ACs, and in whole-cell recordings from W3-RGCs, find that properties of their excitatory input match VG3-AC responses. Finally, we show amplitude and object motion preference of synaptic excitation and spike responses of W3-RGCs are reduced in VGluT3 knockout mice (*VGluT3*^*−/−*^ mice) (Seal et al., 2008). Thus, we identify VG3-ACs as object motion detectors, characterize the synaptic mechanisms underlying this computation, and show that VG3-ACs provide feature-selective excitatory input to W3-RGCs.

## Results and discussion

**Figure 1.**
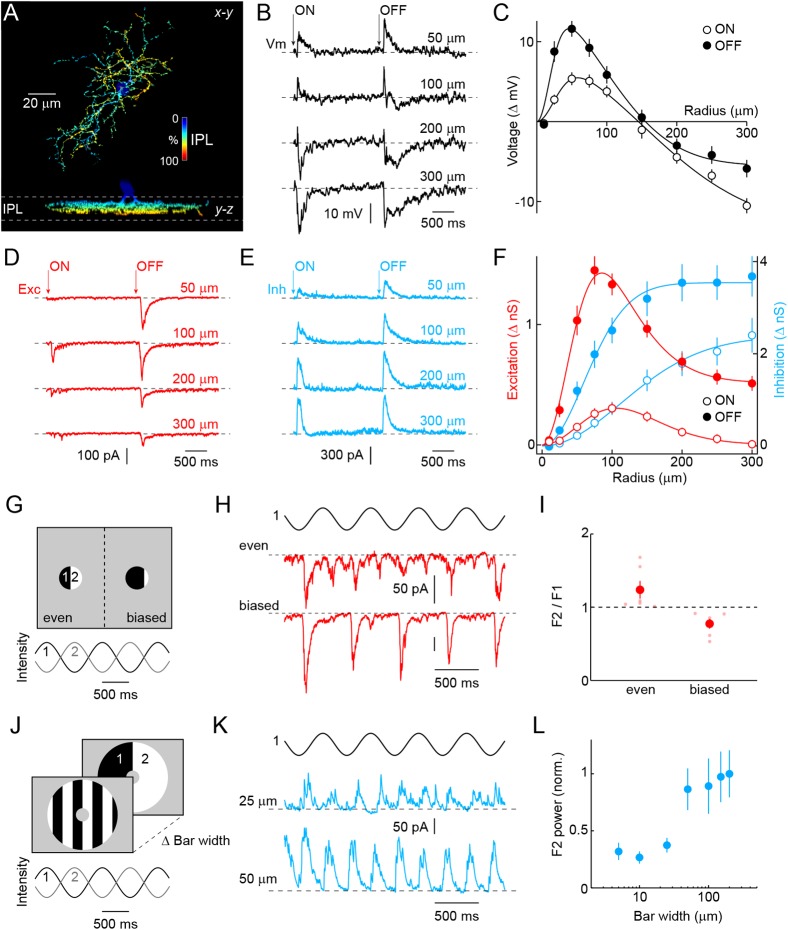
Morphology and receptive field properties of VG3-ACs. (**A**) Orthogonal maximum intensity projections of a confocal image stack through a representative VG3-amacrine cells (ACs) labeled in *VG3-CreERT2 Ai9* mice. The fluorescent signal is colored to reflect depth in the inner plexiform layer (*IPL*). Inset bar graph shows the mean ± SEM territory size of VG3-ACs (n = 39) measured as the area of the smallest convex polygon to encompass their arbors in a z-projection. (**B**, **D**, **E**) Representative voltage (**B**, *black*), excitatory postsynaptic current (EPSC) (**D**, *red*), and inhibitory postsynaptic current (IPSC) (**E**, *blue*) responses to a stimulus in which luminance in a circular area of varying size is square-wave modulated (2 s ON, 2 s OFF, transitions indicated by ‘*arrows*’). Stimuli were presented in pseudorandom order centered on the soma of the recorded cell. Each response trace is annotated with the radius of the stimulus eliciting it. The resting membrane potential of VG3-ACs in our recordings was −38 ± 1.2 mV (n = 26). (**C**, **F**) Summary data of the spatial ON (*open circles*) and OFF (*filled circles*) sensitivity profiles of VG3-ACs for voltage responses (**C**, *black*, n = 26) and excitatory (**F**, *red*, n = 38) and inhibitory (**F**, *blue*, n = 38) conductances. Solid lines show fits of Difference-of-Gaussian (for voltage and excitation) and single Gaussian (for inhibition) models to the data. Receptive field diameters determined from fits to voltage responses were: ON-center 73.4 ± 8.5 μm, OFF-center 40.9 ± 4.2 μm, p < 0.002, ON-surround 290.2 ± 25.5 μm, OFF-surround 213.3 ± 11.3 μm, p < 10^−3^. Receptive field diameters for excitatory inputs were: ON-center 137 ± 15.8 μm, OFF-center 83.1 ± 10.2 μm, p < 0.005, ON-surround 206.4 ± 14.3 μm, OFF-surround 189.8 ± 23.2 μm, p > 0.4. Diameters of inhibitory center-only receptive fields were: ON 258 ± 24.7 μm, OFF 148.2 ± 12.3 μm, p < 10^−4^. Response amplitudes to OFF stimuli exceeded those to ON stimuli for voltage (at 100 μm, p < 10^−9^), excitation (at 100 μm, p < 10^−11^), and inhibition (at 100 μm, p < 10^−5^). (**G**) Schematic illustration of split-field stimuli. The receptive field center is divided evenly (*left*) or in a biased manner (right) into two regions in which intensity is modulated by phase-shifted sine waves. (**H**, **I**) Representative EPSC traces and summary data (n = 6, p < 0.05) for even (*top*) and biased (*bottom*) split-field stimulation. (**J**) Schematic illustration of counter phase stimulation of surround regions. The receptive field surround is divided in bars of different size and their intensity is modulated by phase-shifted sine waves. (**K**) Representative IPSC traces to counter phase stimulation of bars of 25 μm (*middle*) and 50 μm (*bottom*) widths. (**L**) Summary data illustrating change in F2 power of inhibition as a function of bar widths. See also Figure 1—figure supplement 1 and Figure 1—figure supplement 2. **DOI:** http://dx.doi.org/10.7554/eLife.08025.003

To analyze the morphology of VG3-ACs, we generated bacterial artificial chromosome (BAC) transgenic mice expressing a ligand-activated Cre recombinase under control of regulatory sequences of the *Slc17a8* gene (*VG3-CreERT2* mice) and crossed them to a fluorescent reporter strain (*Ai9*) (Madisen et al., 2010). After tamoxifen injection, a subset of VG3-ACs expresses tdTomato in *VG3-CreERT2 Ai9* mice (Figure 1—figure supplement 1). Neurites of VG3-ACs stratify broadly in the center of the inner plexiform layer (Grimes et al., 2011), occupy medium-sized lateral territories (Figure 1A and Figure 1—figure supplement 2, 7662 ± 211 μm^2^, n = 39), and as a population, cover the retina approximately seven times (coverage: 6.88). To characterize light responses, we obtained *VG3-Cre* mice (Grimes et al., 2011) in which all VG3-ACs express Cre (Figure 1—figure supplement 1), crossed them to *Ai9*, and targeted fluorescent somata in the inner nuclear layer (INL) for whole-cell patch-clamp recordings. Consistent with previous results, we find that VG3-ACs respond transiently to light increments and decrements, depolarizing to small and hyperpolarizing to large stimuli (Lee et al., 2014) (Figure 1B,C). Voltage-clamp recordings revealed that this switch in response polarity is caused by a combination of pre- and post-synaptic surround inhibition (Figure 1D–F). Excitatory and voltage-response receptive fields are well fit by Difference-of-Gaussians models (Enroth-Cugell and Robson, 1966; McMahon et al., 2004), whereas a single Gaussian is sufficient to describe the monotonic rise of inhibition with stimulus size. For all components, OFF responses exceed ON responses, and for voltage responses and excitatory inputs, ON receptive fields are larger in diameter than their OFF counterparts. We characterized the temporal tuning of VG3-AC responses and underlying synaptic inputs in more detail using white noise stimuli (Figure 1—figure supplement 3).

**Figure 1—figure supplement 1.**
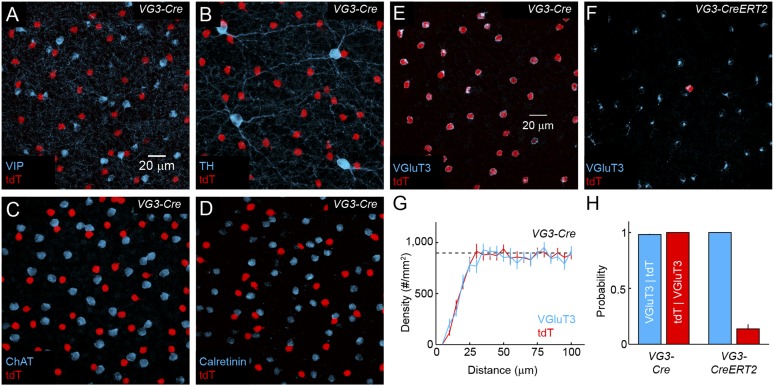
Distribution and specificity of *VG3-Cre* and *VG3-CreERT2* labeling. (**A**–**F**) Maximum intensity projections of confocal image stacks from the INL of *VG3-Cre* mice crossed to a reporter strain expressing the red fluorescent protein tdTomato (*Ai9*, *tdT*, **A**–**E**) stained for vasoactive intestinal peptide (*VIP*, **A**), tyrosine hydroxylase (*TH*, **B**), choline acetyltransferase (*ChAT*, **C**), Calretinin (**D**) and VGluT3 (**E**), and of *VG3-CreERT2 Ai9* mice stained for VGluT3 (**F**). (**G**) Density recovery profiles for tdTomato (n = 17 retinas) and VGluT3 (n = 10 retinas) signals in images like that shown in (**A**) from *VG3-Cre Ai9* mice showed that VG3-ACs are arranged in regular mosaics with characteristic exclusion zones in their density recovery profiles (average densities VGluT3: 898 ± 34 cells/mm^2^, n = 10 retinas, tdTomato: 948 ± 37 cells/mm^2^, n = 17 retinas, effective radii of exclusion zones VGluT3: 18.1 ± 1.8 μm, tdTomato: 16.9 ± 0.4 μm) (Rodieck, 1991). The density of VG3-ACs was not significantly different between dorsal, ventral, nasal, and temporal quadrants of the retina (*data not shown*). (**H**) Conditional probabilities illustrating the specificity (*blue bars*) and completeness (*red bars*) of genetic labeling in *VG3-Cre* and *VG3-CreERT2* mice. While Cre expression is highly specific in the INL, ectopic expression was observed in a small subset of cells in the ganglion cell layer (GCL) in VG3-Cre (Figure 4) but not *VG3-CreERT2* mice. **DOI:** http://dx.doi.org/10.7554/eLife.08025.004

**Figure 1—figure supplement 2.**
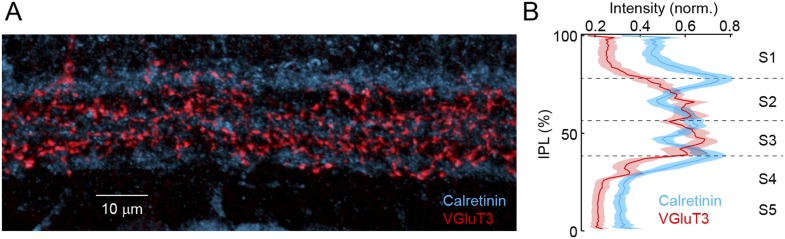
VG3-ACs stratify in sublaminae 2 and 3 of the IPL. (**A**) Representative maximum intensity projection of a confocal image stack of the IPL acquired in a retinal vibratome slice stained for Calretinin (*blue*) and VGluT3 (*red*). Inner nuclear and GCLs are bordering the top and bottom, respectively, of this image. (**B**) Intensity profiles (mean ± SEM, n = 10 retinas) of these labels show that VG3-ACs stratify in sublamina 2 and 3 (Figure 1—figure supplement 2 and Figure 1—figure supplement 3) of the IPLs, the boundaries of which are marked by Calretinin (Wassle, 2004). Note the potential strategic importance of this laminar position. We find that OMS responses of VG3-ACs depend on convergent input from transient rectified ON and OFF bipolar cells. Figure 1—figure supplement 2 and Figure 1—figure supplement 3 contain terminals of OFF and ON bipolar cells, respectively (Wassle et al., 2009; Helmstaedter et al., 2013). Furthermore, bipolar cells with transient responses stratify near the center of the IPL (i.e., Figure 1—figure supplement 2 and Figure 1—figure supplement 3), whereas axons of bipolar cells with sustained responses stratify closer to its borders (Roska and Werblin, 2001; Baden et al., 2013; Borghuis et al., 2013). Finally, a recent study identified gradients in the linearity of glutamate release across the IPL with more rectified bipolar cells stratifying towards the middle (Borghuis et al., 2013). Stratification in Figure 1—figure supplement 2 and Figure 1—figure supplement 3, thus, positions VG3-ACs ideally to recruit input from transient rectified ON and OFF bipolar cells, as well as to form synapses with dendrites of W3-RGCs. The strategic importance of this laminar position is further corroborated by the observation that in all species examined, VG3-ACs and LED RGCs stratify near the center of the IPL (Berson et al., 1998; Roska and Werblin, 2001; Famiglietti, 2005; van Wyk et al., 2006; Zhang et al., 2012). **DOI:** http://dx.doi.org/10.7554/eLife.08025.005

**Figure 1—figure supplement 3.**
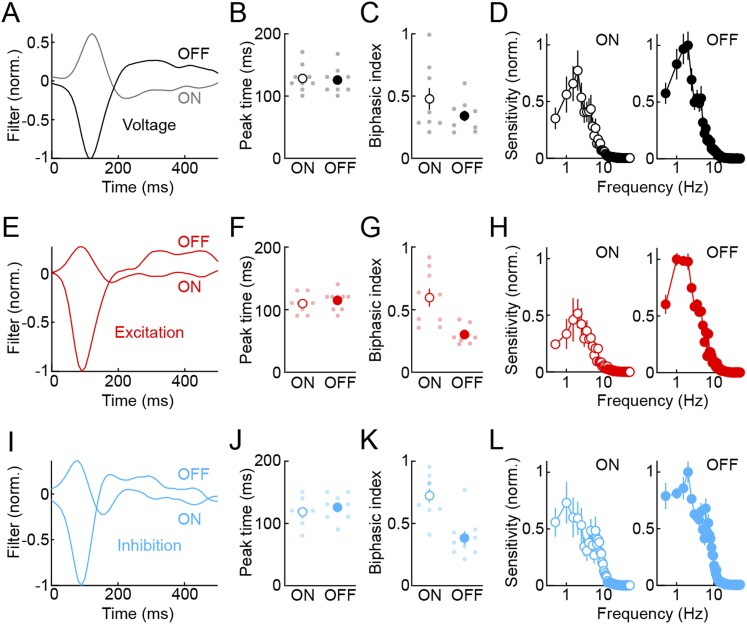
Temporal receptive fields of VG3-ACs. To characterize temporal receptive fields of VG3-ACs, we presented white noise stimuli (refresh rate: 30 Hz, RMS contrast: 40%) to receptive field centers (voltage and excitation) or surrounds (inhibition) and adapted a principal-component-based approach to recover linear filters describing temporal sensitivity to ON and OFF stimuli, respectively (‘Materials and methods’) (Greschner et al., 2006; Gollisch and Meister, 2008). (**A**, **E**, **I**) Linear ON and OFF filters constructed from voltage (**A**, *black*), excitation (**E**, *red*), and inhibition (**I**, *blue*) traces of representative VG3-ACs. (**B**, **C**, **F**, **G**, **J**, **K**) Peak times (**B**, **F**, **J**) and biphasic indices (**C**, **G**, **K**, ON: |trough|/peak, OFF: peak/|trough|) of ON and OFF filters. Dots show data from individual cells and circles (error bars) indicate mean (± SEM) of the population. Peak times of ON and OFF filters were not significantly different for voltage (**B**, *black*, n = 9, p > 0.2), excitation (**F**, *red*, n = 9, p > 0.4), and inhibition (**J**, *blue*, n = 9, p > 0.08). However, ON filters were more biphasic than OFF filters for excitation (**G**, *red*, p < 0.002) and inhibition (**K**, *blue*, p < 0.002), but not voltage responses (**B**, *black*, p > 0.1). (**D**, **H**, **L**) Temporal frequency tuning functions calculated from Fourier amplitudes of ON (*left panels*) and OFF (*right panels*) filters for voltage (**D**, *black*), excitation (**H**, *red*), and inhibition (**L**, *blue*) responses show response suppression at high- and low-stimulus frequencies and illustrate the higher sensitivity of VG3-ACs and their synaptic inputs to OFF compared to ON stimuli. Circles (error bars) show mean (± SEM) of the population. **DOI:** http://dx.doi.org/10.7554/eLife.08025.006

The arbor size of VG3-AC neurites suggests that they receive input from >50 bipolar cells, which each sample a smaller region of visual space (Wassle et al., 2009; Morgan and Kerschensteiner, 2011). Third order neurons that receive convergent rectified input from bipolar cells can detect motion and other changes in patterns with structure on the scale of bipolar cells' receptive fields even when the average luminance across their own receptive fields does not change (i.e., nonlinear spatial integration) (Victor and Shapley, 1979; Demb et al., 2001; Schwartz et al., 2012). To evaluate input rectification of VG3-ACs, we presented sinusoidally modulated split-field stimuli in which the receptive field center was divided evenly or in a biased manner (Figure 1G). We then compared the power of excitatory postsynaptic current (EPSC) responses at once (F1) and twice (F2) the frequency of modulation (2 Hz, Figure 1H,I). Excitatory input to biased split-field stimuli is modulated primarily at the stimulus frequency and recapitulates the OFF-preference of VG3-ACs. The F2-dominant responses to even split-field stimulation indicate that this excitatory input is provided by rectified bipolar cells (Grimes et al., 2014). To test whether inhibition, like excitation, is driven by rectified receptive field subunits and to measure their spatial extent, we sinusoidally modulated square-wave gratings with different bar widths in an annular region overlaying the receptive field surround (Figure 1J). Inhibitory postsynaptic currents (IPSCs) show frequency-doubled (F2) responses characteristic of rectified input (Figure 1K,L). F2 power increases in a step-like fashion between bar widths of 25 μm and 50 μm, suggesting that bipolar cells are likely the cellular substrate for nonlinear subunits (Victor and Shapley, 1979; Demb et al., 2001; Schwartz et al., 2012). Thus, VG3-ACs receive rectified excitatory input from transient ON and OFF bipolar cells, and inhibition from ACs, which themselves appear to be driven by rectified input from possibly the same types of bipolar cells.

**Figure 2.**
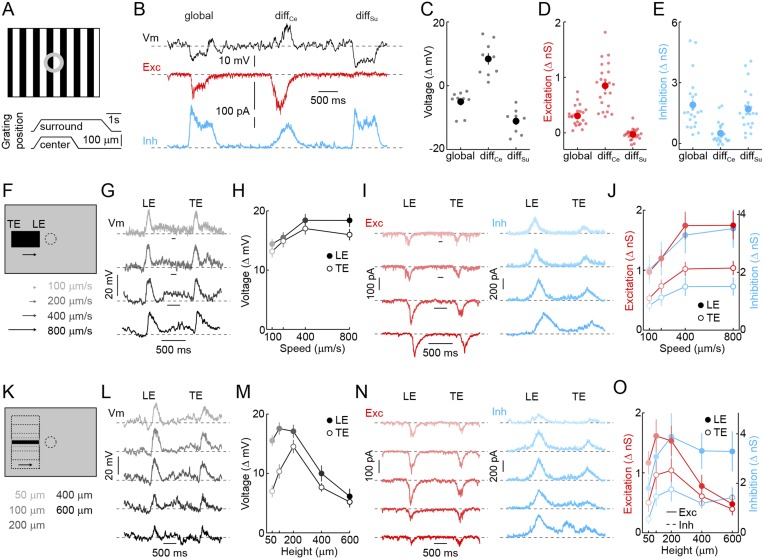
Detection of object motion by VG3-ACs. (**A**) Schematic illustrating texture motion stimuli. Two square-wave gratings (bar width: 50 μm), one covering the center and one the surround region of the VG3-AC receptive field, are separated by a gray annulus. During stimulus presentation, both gratings move first together (*global*) and then separately (differential center motion denoted by *diff*_*Ce*_ and differential surround motion by *diff*_*Su*_). (**B**) Representative voltage (*black*), EPSC (*red*), and IPSC (*blue*) traces recorded during presentation of the stimulus shown in (**A**). (**C**–**E**) Summary data of voltage (**C**), excitatory (**D**), and inhibitory (**E**) response amplitudes to *global*, differential center (*diff*_*Ce*_), and differential surround (*diff*_*Su*_) motion stimuli. Dots show data from individual cells (voltage n = 9, excitation n = 22, inhibition n = 22, p < 10^−7^ for all comparisons), and circles (error bars) indicate mean (±SEM) of the respective population. (**F**) Schematic illustrating a stimulus in which a narrow bar (height: 200 μm) is moved across the receptive field of a VG3-AC at a variety of speeds annotated in (**F**) and encoded by color saturation throughout. (**G**, **I**) Representative voltage (**G**, *black*), EPSC (**I**, *red*), and IPSC (**I**, *blue*) traces recorded in response to bars moving at different speeds. Time points when the leading (*LE*) and trailing edges (*TE*) of the bar are in the center of the receptive field are indicated. (**H**, **J**) Summary data of voltage (**H**, *black*), excitation (**J**, *red*), and inhibition (**J**, *blue*) response amplitudes. Circles (error bars) indicate the mean (±SEM) of these data sets (voltage n = 32, excitation n = 8, inhibition n = 5). (**K**) Schematic illustrating a stimulus in which bars of varying height are moved across the receptive field of a VG3-AC at a constant speed (400 μm/s). Bar heights are encoded in by color saturation as indicated in (**K**). (**L**–**O**) Representative voltage (**L**, *black*), EPSC (**N**, *red*), and IPSC (**N**, *blue*) traces. Summary data of voltage (**M**), excitation (**O**, *red*), and inhibition (**O**, *blue*) response amplitudes reveal suppression of edge responses for voltage and excitation, but not inhibition at greater bar heights (comparison of 200 μm and 600 μm, voltage n = 32, p < 10^−7^ excitation n = 8, p < 0.001, inhibition n = 5, p > 0.14). Circles (error bars) indicate the mean (±SEM) of these data sets. See also Figure 1—figure supplement 3. **DOI:** http://dx.doi.org/10.7554/eLife.08025.007

The receptive field mechanisms described so far—convergence of transient ON and OFF inputs, strong surround inhibition, and rectified receptive field subunits—led us to hypothesize that VG3-ACs may selectively detect the movements of small objects. To test this hypothesis, we evaluated voltage responses and synaptic inputs of VG3-ACs in differential motion (Baccus et al., 2008; Zhang et al., 2012) and edge detection stimulus paradigms (Levick, 1967; van Wyk et al., 2006). When square-wave gratings overlaying center and surround regions of their receptive field were shifted separately or together (Figure 2A) (Baccus et al., 2008; Zhang et al., 2012), VG3-ACs depolarized robustly to differential motion in the center, but hyperpolarized to synchronous movements in center and surround (i.e., global motion) and differential motion in the surround (Figure 2B,C). Voltage-clamp recordings revealed that this response pattern is caused by preferential excitation during center-only motion and strong inhibition elicited whenever motion includes the surround (Figure 2B,E,D). Since the average luminance in center and surround regions does not change in this stimulus, the observed responses provide further evidence that both are composed of rectified subunits, allowing VG3-ACs to detect when an object moves at a different time than the background irrespective of the precise spatial patterns involved, a defining feature of OMS neurons (Olveczky et al., 2003; Baccus et al., 2008).

**Figure 2—figure supplement 1.**
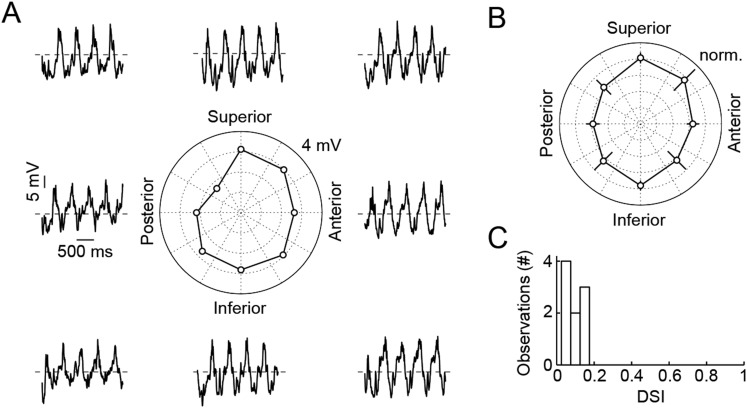
Responses of VG3-ACs are not direction selective. (**A**) Representative responses of a VG3-AC to a sine grating drifting in eight different directions (frequency: 2 Hz). The polar plot in the center shows the Fourier amplitude of the response at the stimulus frequency (F1). (**B**) Polar plot summarizing responses of nine VG3-ACs (mean ± SEM). (**C**) Histogram of direction selectivity index (DSI) values of these VG3-ACs. The DSI was calculated as the absolute amplitude of $\frac{\sum F1\left(\theta \right)e^{i\theta }}{\sum F1\left(\theta \right)}$. **DOI:** http://dx.doi.org/10.7554/eLife.08025.008

Next, we tested the ability of VG3-ACs to detect contrast edges. Narrow dark bars moving across the receptive fields of VG3-ACs elicited transient depolarizations as leading and trailing edges cross the receptive field center. Robust edge responses were observed over a wide range of motion speeds (Figure 2G,H) and were indistinguishable for leading and trailing edges in spite of the opposite polarity of the associated temporal contrast. Responses of VG3-ACs are not directionally selective (Figure 2—figure supplement 1). Similar to voltage recordings, transient EPSCs and IPSCs were observed during edge transits (Figure 2I). EPSCs and IPSCs were larger at leading than trailing edges and showed a preference for the faster speeds tested (Figure 2J). However, the ratio of excitation and inhibition remained relatively constant across these conditions, which likely accounts for the greater constancy of edge responses observed in voltage recordings. Local edge detectors are typically stimulated robustly by narrow contrast edges, but not by expansive ones (Levick, 1967; Roska and Werblin, 2001; Zeck et al., 2005; van Wyk et al., 2006). We, therefore, recorded responses of VG3-ACs to bars of varying heights moving across their receptive field at a constant speed (Figure 2K). Edge responses were strongly suppressed for bar heights above 200 μm (Figure 2L,M). Trailing edge responses to the smallest bars were lower than leading edge responses, likely a consequence of the asymmetric size of VG3-ACs' ON and OFF receptive field centers (Figure 1). Similar to voltage responses, excitatory synaptic inputs showed edge response suppression for larger stimuli, whereas inhibition rose and plateaued with increasing bar height (Figure 2N,O), corroborating that surround inhibition acts both pre- and post-synaptic in VG3-ACs. VG3-ACs, thus, show key response features of OMS and local-edge-detector neurons, and through the synaptic mechanisms outlined above, selectively detect movements of small objects.

**Figure 3.**
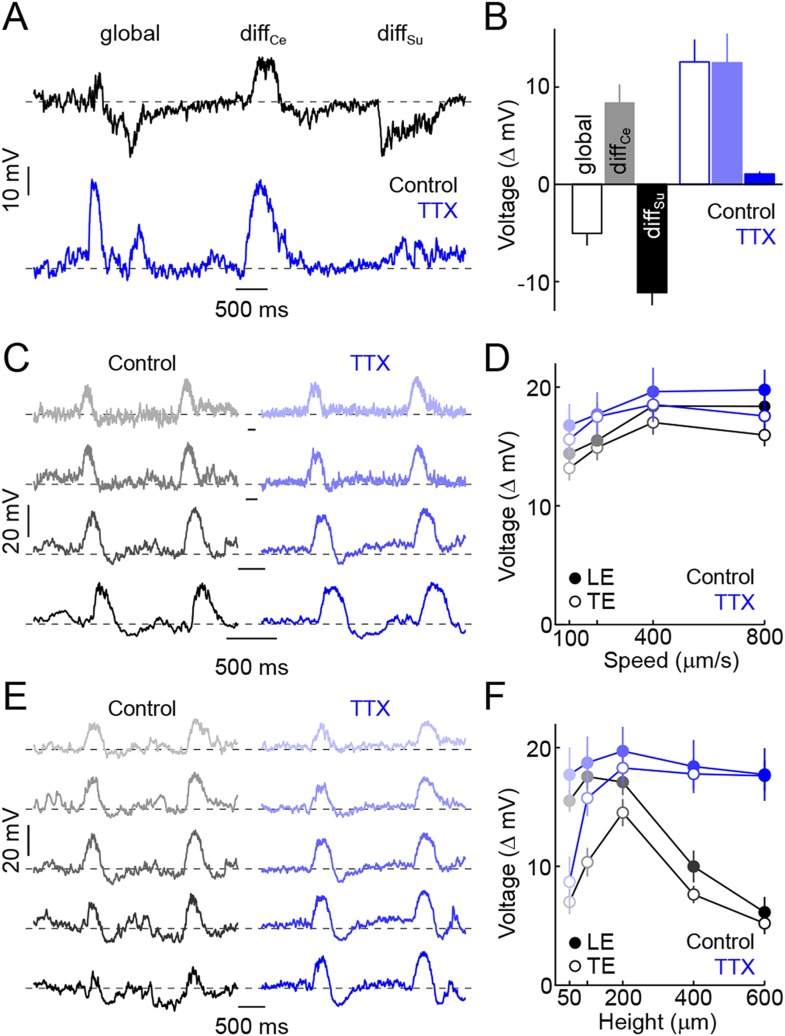
Spiking ACs mediate surround suppression of VG3-ACs. (**A**) Representative voltage traces recorded from a VG3-AC during presentation of the texture motion stimulus illustrated in Figure 2A in control conditions (*top, black*) and in the presence of TTX (*bottom, blue*). (**B**) Bars (error bars) indicating mean (±SEM) response amplitudes to different segments of the texture motion stimulus in control conditions (*left, black*) and in the presence of TTX (*right, blue*). TTX abolishes the suppression by global and differential surround motion (control n = 9, TTX n = 5, p < 10^−4^ for TTX vs control) but does not affect the response to differential center motion (p > 0.2 for TTX vs control). (**C**, **D**) Representative traces (**C**) and summary data (**D**, mean ± SEM) for voltage responses of VG3-ACs to narrow dark bars (height: 200 μm) moving at different speeds (100 μm/s, 200 μm/s, 400 μm/s, and 800 μm/s from *top* to *bottom*), encoded by matching color saturation in (**C**) and (**D**) in the absence (*left, black*) or presence (*right, blue*) of TTX. TTX did not significantly alter responses to narrow bars irrespective of their speed (control n = 32, TTX n = 7, p > 0.1 for all comparisons). (**E**, **F**) Exemplary traces (**E**) and population data (**F**, mean ± SEM) for voltage responses of VG3-ACs to dark bars of different heights (50 μm, 100 μm, 200 μm, 400 μm, and 600 μm from *top* to *bottom*) moving at 400 μm/s, indicated by matching color saturation in (**E**) and (**F**), in the absence (*left, black*) or presence (*right, blue*) of TTX. Surround suppression is canceled by TTX leading to increased responses to larger bars (at 600 μm, control n = 32, TTX n = 7, p < p < 10^−4^). **DOI:** http://dx.doi.org/10.7554/eLife.08025.009

The data presented so far suggest that pre- and post-synaptic surround inhibition cancel responses of VG3-ACs to global scene shifts and movements of large objects. Surround suppression of the excitatory input to W3-RGCs was found to rely on spiking ACs (Zhang et al., 2012). To test whether surround suppression of VG3-ACs is similarly mediated by spiking ACs and to assess its importance to feature detection, we tested the effect of the sodium channel blocker TTX on VG3-ACs' responses in differential motion and edge detection stimulus paradigms. In the presence of TTX, VG3-ACs depolarized to global motion as well as differential center motion, and hyperpolarizations observed during surround stimulation were abolished (Figure 3A,B). Edge responses elicited by narrow bars moving across the receptive fields of VG3-ACs at a variety of speeds were not changed by TTX (Figure 3C,D), but the suppression observed for larger bars was blocked (Figure 3E,F). Surround inhibition, thus, is critical for the feature detection of VG3-ACs and appears to be mediated by spiking ACs. The use of spikes to signal surround motion likely improves temporal coincidence of inhibitory input with bipolar cell depolarization and excitatory input to VG3-ACs during coherent motion, leading to more effective cancellation of center signals by the surround.

**Figure 4.**
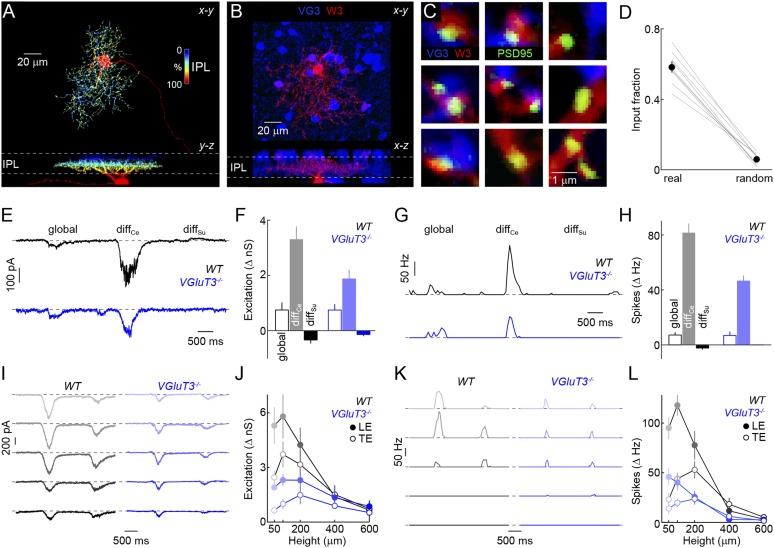
Anatomy and function of input from VG3-ACs to W3-RGCs. (**A**) Orthogonal projections of a confocal image stack through a representative W3-retinal ganglion cell (RGC) labeled biolistically with cyan fluorescent protein (CFP). W3-RGCs were identified by their characteristic morphology (Kim et al., 2010; Zhang et al., 2012) with small dendritic fields (territory size: 10,783 ± 409 μm^2^, n = 25) filled by densely branched neurites stratifying in the center of the IPL with a secondary arborization near the border between the inner plexiform and inner nuclear layers (INLs). The fluorescent signal is colored to represent depth in the *IPL*. Inset bar graph shows the mean ± SEM territory size of W3-RGCs (n = 15) measured as the areas of the smallest convex polygons to encompass their arbors in a z-projection. (**B**, **C**) Overview projections (**B**) and single plane excerpts (**C**) of a W3-RGC biolistically labeled with CFP (*red*) and PSD95-YFP (*green*) in a *VG3-Cre Ai9* mouse (tdTomato shown in *blue*). (**D**) Summary data indicating the fraction of PSD95-YFP puncta apposed by VG3-AC boutons (‘Materials and methods’) in the obtained images (*real*) or when positions of PSD95-YFP puncta were randomized within the synaptic layer (*random*) in Monte Carlo simulations (n = 9 cells, p < 10^−6^). Gray lines indicate data from individual cells; circles (error bars) show the mean (±SEM) of the population. (**E**–**H**) Representative EPSC (**E**) and spike response (**G**) traces to the texture motion stimulus illustrated in Figure 2A recorded from W3-RGCs (wild-type [*WT*] *black*, vesicular glutamate transporter 3 [*VGluT3*^*−/−*^] *blue*), and bar plots summarizing differences in excitatory conductance (**F**) and spike rates (**H**) during different segments of the stimulus in *WT* (*left, black*) and *VGluT3*^*−/−*^ mice (*right, blue*). Bars (error bars) indicate the mean (±SEM) of the respective data sets. W3-RGC EPSCs in *VGluT3*^*−/−*^ mice were significantly reduced compared to *WT* littermates during differential center motion (*WT* n = 8, *VGluT3*^*−/−*^ n = 9, p < 0.02), but not global or differential surround motion (p > 0.1 for both). A similar pattern was observed in the spike responses of W3-RGCs, which were decreased for differential center motion (*WT* n = 13, *VGluT3*^*−/−*^ n = 9, p < 0.001), but not altered during global image motion (p > 0.9). (**I**–**L**) Representative EPSC (**I**) and spike response (**K**) traces, and summary data (excitation in **J**, spikes in **L**, mean ± SEM) recorded in W3-RGCs during stimulation with dark bars of different heights (indicated by color saturation) moving at 400 μm/s in *WT* (*left, black*) and *VGluT3*^*−/−*^ mice (*right, blue*). Whereas excitatory inputs and spike responses were reduced for narrow bars (excitation at 100 μm, *WT* n = 6, *VGluT3*^*−/−*^ n = 6, p < 0.03, spikes at 100 μm, *WT* n = 14, *VGluT3*^*−/−*^ n = 8, p < 0.01), they did not differ significantly for bars of greater heights (excitation at 600 μm, *WT* n = 6, *VGluT3*^*−/−*^ n = 6, p > 0.6, spikes at 600 μm, *WT* n = 14, *VGluT3*^*−/−*^ n = 8, p < 0.2). See also Figure 2—figure supplement 1, Figure 4—figure supplement 1 and Figure 4—figure supplement 2. **DOI:** http://dx.doi.org/10.7554/eLife.08025.010

We next wondered how signals of VG3-ACs are used in the retina. Recent optogenetic experiments and paired recordings suggest that VG3-ACs can release glutamate and activate postsynaptic receptors on several RGC types, including W3-RGCs (Lee et al., 2014). Whether and how VG3-ACs contribute to visual processing in these RGCs remains unknown. Given our results on object-motion detection, we focus here on VG3-ACs' connections with and influence on W3-RGCs. To obtain anatomical evidence for or against excitatory synapses between VG3-ACs and W3-RGCs, we biolistically labeled W3-RGCs with cytosolic cerulean fluorescent protein and PSD95 fused to yellow fluorescent protein (PSD95-YFP) in *VG3-Cre Ai9* mice (Figure 4A,B). PSD95-YFP selectively localizes to excitatory synapses on RGC dendrites (Morgan et al., 2008; Kerschensteiner et al., 2009). More than half of the PSD95-YFP puncta on W3-RGCs were apposed by VG3-ACs boutons, whereas few appositions with VG3-ACs were observed when PSD95-YFP puncta were randomly repositioned along the dendrites in Monte Carlo simulations (Figure 4C,D). We next characterized spike responses and synaptic inputs of W3-RGCs with the same differential motion and edge detection stimuli used for VG3-ACs, revealing matching tuning properties of excitatory input to W3-RGCs with responses of VG3-ACs (Figure 4—figure supplement 1).

**Figure 4—figure supplement 1.**
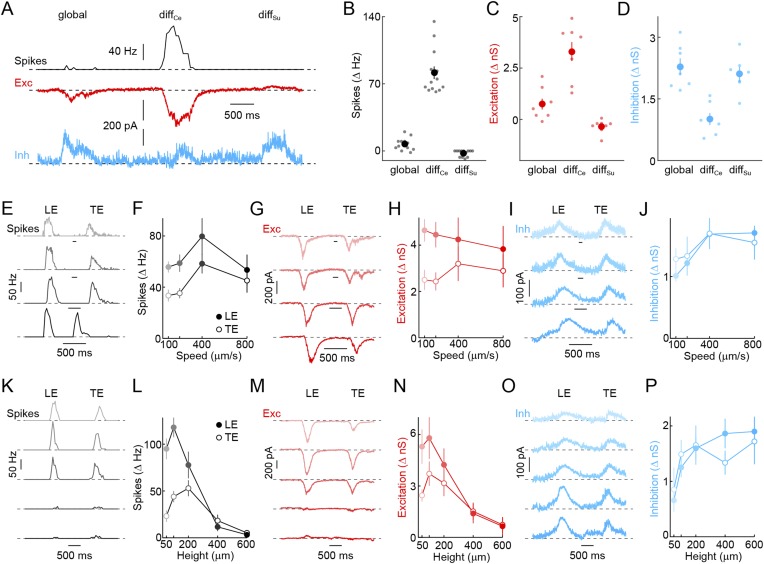
Detection of object motion by W3-RGCs. (**A**) Representative spike rate (*black*), EPSC (*red*), and IPSC (*blue*) traces recorded from W3-RGCs during presentation of the texture motion stimuli (illustrated in Figure 2A). W3-RGCs were either recorded under conventional infrared illumination and identified by characteristic responses in cell-attached recordings or targeted under 2-photon guidance in *Isl2*-*GFP* transgenic mice (Triplett et al., 2014). In both cases, correct targeting was confirmed by intracellular dye filling and reconstruction of dendritic arborizations at the end of the recordings. (**B**–**D**) Summary data of spike rate (**B**, *black*), excitatory (**C**, *red*), and inhibitory (**D**, *blue*) response amplitudes to *global*, differential center (*diff*_*Ce*_), and differential surround (*diff*_*Su*_) motion segments. Dots show data from individual cells and circles (error bars) indicate mean (± SEM) of the respective population. W3-RGCs remain mostly silent during global and differential surround motion stimulation, but show robust spike responses to grating movements restricted to their receptive field center (n = 13, p < 10^−7^ for *diff*_*Ce*_ vs *global* and vs *diff*_*Su*_). Differential motion sensitivity of WG3-RGCs appears to be inherited from their excitatory input, which during center-only motion exceeds that observed during global motion nearly fivefold, and which is suppressed from tonic levels during isolated surround stimulation (n = 8, p < 0.003 for *diff*_*Ce*_ vs *global* and vs *diff*_*Su*_). In addition, W3-RGCs receive stronger direct inhibition when grating movements include the surround (n = 7, p < 0.001 for *diff*_*Ce*_ vs *global* and vs *diff*_*Su*_) (**E**–**J**) Representative spike rate (**E**, *black*), EPSC (**G**, *red*), and IPSC (**I**, *blue*) traces and summary data of spike (**F**, *black*), excitatory (**H**, *red*), and inhibitory (**J**, *blue*) response amplitudes to dark bars (height: 200 μm) moving at different speeds indicated by matching color saturation of example traces and summary data. Circles (error bars) represent the mean (± SEM) of these data sets (spikes n = 32, excitation n = 8, inhibition n = 5). (**K**–**P**) Representative spike rate (**K**, *black*), EPSC (**N**, *red*), and IPSC (**O**, *blue*) responses elicited by bars of different heights moving at 400 μm/s and summary data of spike rate (**L**, *black*), excitatory (**N**, *red*), and inhibitory (**P**, *blue*) response amplitudes. Bar heights are encoded by matching color saturation of responses traces and summary data. Circles (error bars) represent the mean (± SEM) of these data sets. Responses of W3-RGCs are progressively suppressed when bar heights increase above 100 μm (n = 14, p < 10^−4^ for 200 μm vs 600 μm bar heights). The spatial tuning of the excitatory input to W3-RGCs is similar to that observed in spike responses (n = 6, p < 0.02 for 200 μm vs 600 μm bar heights), whereas inhibition rises monotonically and saturates with increasing bar heights (n = 6, p > 0.4 for 200 μm vs 600 μm bar heights). **DOI:** http://dx.doi.org/10.7554/eLife.08025.011

**Figure 4—figure supplement 2.**
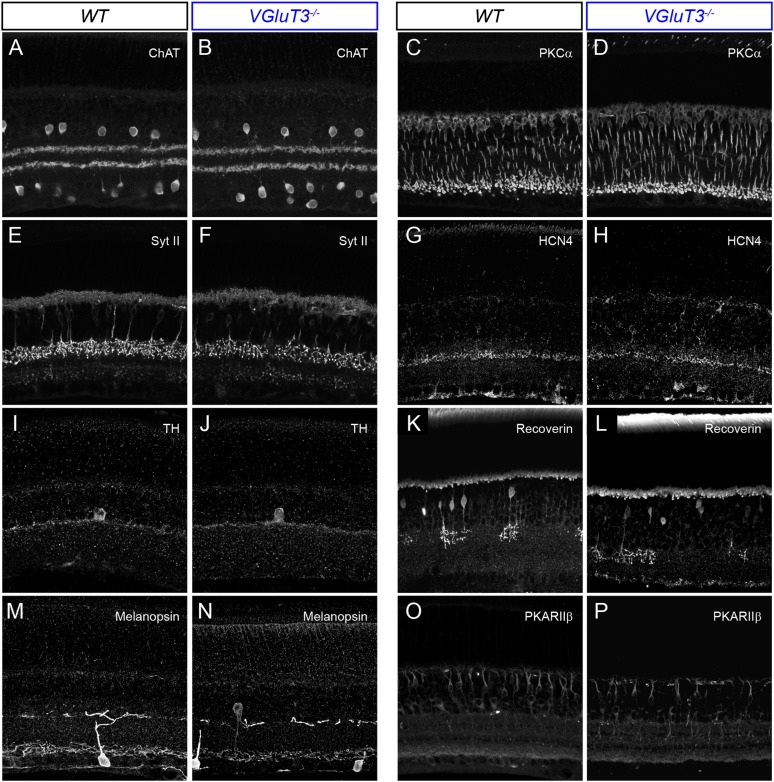
Lamination patterns of cells and neurites are preserved in *VGluT3*^*−/−*^ mice. (**A**–**P**) Maximum intensity projections of confocal image stacks of representative vibratome sections of WT (**A**, **C**, **E**, **G**, **I**, **K**, **M**, **O**) and *VGluT3*^*−/−*^ (**B**, **D**, **F**, **H**, **J**, **L**, **N**, **P**) retinas. Sections were stained for choline acetyltransferase (*ChAT*, **A**, **B**), protein kinase C alpha (*PKCα*, **C**, **D**), synaptotagmin II (*Syt II*, **E**, **F**), hyperpolarization activated cyclic nucleotide gated potassium channel 4 (*HCN4*, **G**, **H**), Tyrosine hydroxylase (*TH*, **I**, **J**), Recoverin (**K**, **L**), Melanopsin (**M**, **N**), and Protein Kinase A regulatory subunit II beta (*PKARIIβ*, **O**, **P**). **DOI:** http://dx.doi.org/10.7554/eLife.08025.012

**Figure 4—figure supplement 3.**
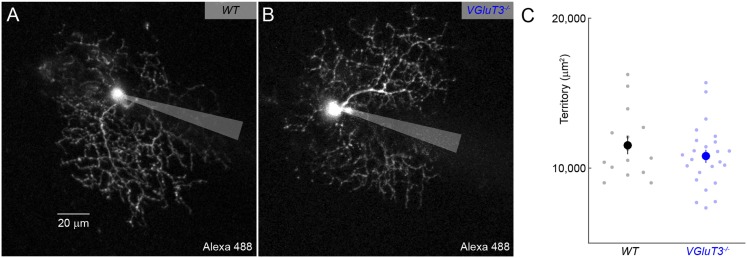
Dendritic morphology of W3-RGCs is unchanged in *VGluT3*^*−/−*^ mice. (**A**, **B**) Maximum intensity projections through 2-photon image stacks of representative W3-RGCs recorded in *WT* (**A**) and *VGluT3*^*−/−*^ (**B**) mice. (**C**) Summary data of dendritic territories covered by W3-RGCs labeled biolistically or filled during patch-clamp recordings in *WT* (*black*) or *VGluT3*^*−/−*^ (*blue*) retinas. **DOI:** http://dx.doi.org/10.7554/eLife.08025.013

**Figure 4—figure supplement 4.**
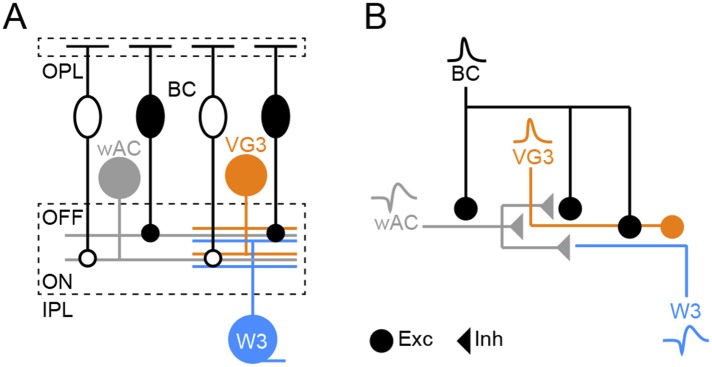
Schematic of object motion detection circuit. (**A**) Overview illustration of the object motion detection circuit. Somata of ON and OFF BCs are shown as open and filled ovals, respectively. Axons of these neurons converge onto the other components of the circuit: wide-field ACs (wACs), VG3-ACs, and W3-RGCs. (**B**) Schematic of the inferred connectivity motif, repeated in ON and OFF layers of the object motion detection circuit. Excitatory and inhibitory synaptic output is shown by circles and triangles, respectively, and use of spikes (*wAC*, *ss*) or graded potentials (*BC*, *VG3*) is indicated by different waveforms. **DOI:** http://dx.doi.org/10.7554/eLife.08025.014

To test whether VG3-ACs provide excitatory input to W3-RGCs during visual stimulation, to compare the tuning of VG3- and non-VG3 inputs, and assess VG3-ACs' contribution to object motion signals sent to the brain, we recorded W3-RGCs in mice lacking VGluT3 (*VGluT3*^*−/−*^ mice) (Seal et al., 2008). Removal of VGluT3, which in the retina is only expressed by VG3-ACs, affected neither gross morphological development of the retina (Figure 4—figure supplement 2) nor dendritic patterns of W3-RGCs (Figure 4—figure supplement 3). EPSCs elicited by differential center motion were reduced by approximately 50% in W3-RGCs of *VGluT3*^*−/−*^ compared to wild-type (*WT*) mice (Figure 4E,F). By contrast, excitation during global motion and tonic excitation revealed by differential surround stimulation were unchanged. A similar pattern was observed in the spike responses of W3-RGCs, which were selectively decreased for differential center but not global motion stimuli (Figure 4G,H). Similarly, EPSCs and spike responses evoked by edges of narrow moving bars (height: 100 μm) were suppressed across a wide range of speeds in *VGluT3*^*−/−*^ mice (Figure 4I–L and *data not shown*), whereas excitation and spike responses elicited by broader moving bars remained intact in *VGluT3*^*−/−*^ mice (Figure 4I–L). In agreement with anatomical results (Figure 4B,C), VG3-ACs, thus, appear to provide approximately half of the excitatory input to W3-RGCs. Importantly, feature selectivity of this VG3-input is more sharply tuned than the excitatory input remaining in *VGluT3*^*−/−*^ mice—likely provided by ON and OFF bipolar cells—and is required for normal spike responses of W3-RGCs.

In the OMS circuit (Figure 4—figure supplement 4), VG3-ACs serve to amplify and sharpen the tuning of responses to object motion. Multi-tiered inhibition combined with delayed excitation, and successive threshold nonlinearities likely contribute to sharpening. Surround inhibition acts at three levels: bipolar axon terminals, VG3-ACs, and W3-RGCs (Zhang et al., 2012; Lee et al., 2014). Key features—transient ON and OFF input driven by rectified subunits—are similar at all three stages, arguing that inhibition is provided by a single AC type or a shared set of AC types, which remain to be identified. The added level of inhibition onto VG3-ACs compared to conventional pathways through bipolar cells likely contributes to the more complete surround suppression in the OMS circuit. Moreover, channeling of excitation through VG3-ACs introduces a delay not shared by the inhibitory input, which could improve cancellation of center signals by the surround, for example, during global image motion. The sequential arrangement of three thresholding nonlinearities—glutamate release from bipolar cells, glutamate release from VG3-ACs, and spike generation in W3-RGCs—likely further contributes to the increasing selectivity for narrow vs broad edges and differential center vs global texture motion at successive stages of the OMS circuit. Finally, our results support the notion that the diversity of AC types and circuit motifs in which they participate are integral to the diversity of features encoded in the signals the retinal sends to the brain (Masland, 2012; Jadzinsky and Baccus, 2013).

## Materials and methods

### Mice

We used homologous recombination to introduce the CreERT2 DNA recombinase into a BAC containing regulatory sequences of the *Slc17a8* gene, which encodes the VGluT3. The resulting construct was injected into pronuclei to generate *VG3-CreERT2 mice*. To induce Cre-mediated recombination in *VG3-CreERT2* mice, adult animals (i.e., older than P21) were injected intraperitoneally with 1 mg tamoxifen for five consecutive days. Both *VG3-CreERT2* and the noninducible *VG3-Cre* (Grimes et al., 2011) mice were crossed to a fluorescent reporter strain expressing tdTomato in a Cre-dependent manner (*Ai9*) (Madisen et al., 2010) to enable anatomical reconstructions and targeted patch-clamp recordings from VG3-ACs. Contributions of VGluT3-mediated neurotransmission to visual processing in the retina were evaluated by comparing knockout mice (*VGluT3*^*−/−*^) (Seal et al., 2008) and *WT* littermates. *Isl2-GFP* transgenic mice (Triplett et al., 2014) were obtained from the Mutant Mouse Regional Resource Center (MMRRC), to which they were donated by Dr Nathaniel Heintz and used for targeted patch-clamp recordings of W3-RGCs.

### Tissue preparation

All procedures in this study were approved by the Animal Studies Committee of Washington University School of Medicine (Protocol #: 20140095) and performed in compliance with the National Institutes of Health *Guide for the Care and Use of Laboratory Animals.* Mice were dark-adapted for >2 hr, deeply anesthetized with CO_2_, killed by cervical dislocation, and their eyes removed. Retinas were isolated and flat mounted on membrane discs (for anatomy: HABG01300, Millipore, Billerica, MA; for physiology: Anodisc 13, Whatman, Pittsburgh, PA). For patch-clamp recordings, enucleation and tissue preparation were carried out under infrared (>900 nm) illumination. For immunohistochemistry, tissue was fixed for 30 min in 4% paraformaldehyde in mouse artificial cerebrospinal fluid (mACSF_HEPES_), washed for >10 min in phosphate-buffered saline (PBS), washed in 10% sucrose in PBS for 1 hr at RT, washed in 20% sucrose in PBS for 1 hr at RT, washed in 30% sucrose in PBS overnight at 4°C, freeze-thawed three times, washed in PBS for >10 min, and incubated in 5% normal donkey serum for 2 hr prior to addition of primary antibodies.

### Immunohistochemistry

Vibratome sections (60 μm thick) and retinal flat mounts were stained with rabbit anti-calretinin (1:1000, Millipore), goat anti-ChAT (1:500, Millipore), rabbit anti-recoverin (1:1000, Millipore), rabbit anti-PKARIIβ (1:500, BD Bioscience, San Jose, CA), rabbit anti-TH (1:1000, Millipore), rabbit anti-HCN4 (1:500, Neuromab, Davis, CA), rabbit anti-VIP (1:1000, Immunostar, Hudson, WI), mouse anti-melanopsin (1:1000, Advanced Targeting Systems, San Diego, CA), mouse anti-PKCα (1:500, Sigma, Saint Louis, MO), and mouse anti-Znp1/SytII (1:1000, ZIRC, Eugene, OR) for 1 (vibratome slices) or 5 days (flat mounts) at 4°C. The tissue was then washed in PBS (3 × 30 min), incubated with DyLight 405- (1:100, Jackson ImmunoResearch, West Grove, PA), Alexa Fluor 488-, Alexa Fluor 568-, and/or Alexa Fluor 633-conjugated secondary antibodies (1:1000, Invitrogen, Grand Island, NY) for 2 hr at RT (vibratome slices) or 2 days at 4°C (flat mounts), washed again in PBS (3 × 30 min), and mounted in Vectashield mounting medium (Vector Laboratories, Burlingame, CA) for confocal imaging.

### Biolistics

Gold particles (1.6-μm diameter, BioRad, Hercules, CA) were coated with plasmids encoding cytosolic cyan fluorescent protein (CFP) and PSD95 fused at its C-terminus to yellow fluorescent protein (PSD95-YFP) (Morgan and Kerschensteiner, 2012). Particles were delivered to RGCs from a helium-pressurized gun (BioRad) at approximately 40 psi (Morgan and Kerschensteiner, 2011). After shooting, retinal flat mount preparations in mACSF_HEPES_—containing (in mM): 119 NaCl, 2.5 KCl, 2.5 CaCl_2_, 1.3 MgCl_2_, 1 NaH_2_PO_4_, 11 glucose, and 20 4-(2-hydroxyethyl)-1-piperazineethanesulfonic acid (HEPES) (pH adjusted to 7.37 with NaOH)—were incubated in a humid oxygenated chamber at 33–35°C for 14–18 hr. The tissue was then fixed for 30 min in 4% paraformaldehyde in mACSF_HEPES_ and washed PBS (3 × 10 min) before mounting and imaging.

### Imaging

VG3-ACs, W3-RGCs, and patterns of connections between them were reconstructed from confocal and 2-photon imaging stacks acquired on Fv1000 laser scanning microscopes (Olympus, Tokyo, Japan) using 60× 1.35 NA oil immersion or 20× 0.9 NA water immersion objectives. Synaptic connectivity was analyzed in image stacks with voxel size 0.103 μm (x/y-axis)–0.3 μm (z-axis), whereas neurite territories were measured in image stacks with voxel size 0.206 μm (x/y-axis)–0.5 μm (z-axis).

### Electrophysiology

Whole-cell patch-clamp recordings from VG3-ACs in the INL and W3-RGCs in the ganglion cell layer were obtained in the dorsal halves (Wei et al., 2010; Wang et al., 2011) of flat-mounted retinas continuously superfused (6–8 ml/min) with warm (33–35°C) mACSF_NaHCO3_ containing (in mM) 125 NaCl, 2.5 KCl, 1 MgCl_2_, 1.25 NaH_2_PO_4_, 2 CaCl_2_, 20 glucose, 26 NaHCO_3_, and 0.5 L-Glutamine equilibrated with 95% O_2_/5% CO_2_. In some experiments, the following pharmacological agents were added to mACSF_NaHCO3_ individually or in combinations (‘Results’) and bath-applied: L-2-Amino-4-phosphonobutyric acid (L-APB, 50 μM, Tocris, Bristol, United Kingdom), 1,2,5,6-tetrahydropyridine-4-yl-methylphosphinic acid (TPMPA, 50 μM, Sigma), gabazine (5 μM, Tocris), strychnine (500 nM, Sigma), and tetrodotoxin (TTX, 1 μM, Sigma). Current-clamp recordings were performed with an intracellular solution containing (in mM): 125 K-gluconate, 10 NaCl, 1 MgCl_2_, 10 ethylene glycol tetraacetic acid (EGTA), 5 HEPES, 5 Adenosine triphosphate disodium salt (ATP-Na_2_), and 0.1 Guanosine triphosphate disodium salt (GTP-Na_2_) (pH adjusted to 7.2 with KOH). The intracellular solution used in voltage-clamp recordings contained (in mM): 120 Cs-gluconate, 1 CaCl_2_, 1 MgCl_2_, 10 Na-HEPES, 11 EGTA, 10 TEA-Cl, and 2 Qx314 (pH adjusted to 7.2 with CsOH). Alexa 488 or 568 were added (0.1 mM) to both intracellular solutions. Patch pipettes had resistances of 4–7 MΩ (borosilicate glass). All reported voltages were corrected for liquid junction potentials. For voltage-clamp recordings, series resistance (10–15 MΩ) was compensated electronically by ∼75%. Signals were amplified with a Multiclamp 700B amplifier (Molecular Devices, Sunnyvale, CA), filtered at 3 kHz (8-pole Bessel low-pass) and sampled at 10 kHz (Digidata 1440A, Molecular Devices). EPSCs were isolated by clamping the voltage of the recorded cell to the reversal potential for Cl^−^ (−60 mV), the main permeant ion of inhibitory transmitter receptors, whereas IPSCs were recorded at the reversal potential of currents through excitatory transmitter receptors (0 mV). In current-clamp recordings, no bias current was injected.

Fluorescent VG3-ACs were targeted under 2-photon guidance (excitation wavelength: 900 nm) in *VG3-Cre Ai9* mice. W3-RGCs were either recorded under conventional infrared illumination (>900 nm) or targeted under 2-photon guidance in *Isl2-GFP* mice. Correct targeting was confirmed by monitoring entry of Alexa dyes (488 or 568) included in the intracellular solution from the recording pipette into the soma during break in and by reconstructing the morphology of neurite arbors at the end of each recording.

### Visual stimulation

Custom stimuli written in MATLAB (The Mathworks, Natick, MA) using Cogent graphics extensions (John Romaya, Laboratory of Neurobiology at the Wellcome Department of Imaging Neuroscience, University College London) were presented on an organic light-emitting display (xOLED, eMagin, Bellevue, WA) and focused onto the photoreceptors through the substage condenser of an integrated 2-photon patch-clamp setup. The average intensity of all stimuli was kept constant at ∼3000 R*/rod/s or ∼2500 M*/M-cone/s and their position centered on the soma of the recorded cell.

To measure area response functions of VG3-ACs and W3-RGCs, the intensity of a circular area with varying radii was square-wave modulated at 0.125 Hz (Michelson contrast: 96%). Circles of different size were presented in different pseudorandom sequences for each cell, and the first stimulus in the sequence repeated at its end to confirm stability of the recording. Temporal response functions and filter properties were analyzed using Gaussian white noise stimulation in which the intensity of a circular region over the receptive field center (voltage, excitation) or an annular region covering its surround (inhibition) was chosen at random from a normal distribution (RMS contrast: 40%) and updated at 30 Hz for 10 min. The properties of spatial integration were tested using sine-wave modulated (2 Hz) contrast-reversing square-wave gratings (varying spatial frequency) masked to preferentially stimulate the center (excitation) and surround (inhibition) regions of a receptive field. To test the sensitivity of VG3-ACs and W3-RGCs to differential or coherent luminance-neutral motion stimuli in their receptive field center and surround, the respective parts of square-wave gratings (bar width: 50–75 μm) were moved separately or in unison. A gray annulus was included in the spatial layout of the stimulus to reliably separate movement in the center and surround. Edge detection properties were tested by moving dark and light bars of different heights (50–600 μm) and constant width (800 μm) across the receptive fields of VG3-ACs and W3-RGCs at a variety of speeds (100–800 μm/s). The order in which bars of different contrast, height, and speed were shown was randomized for each cell.

### Analysis

Data were analyzed using programs written in MATLAB. Response amplitudes to a variety of stimuli—circles of varying size and contrast, bars of different size and contrast moving at a variety of speeds and differential or global motion of gratings—were measured as baseline-subtracted averages (spike rate, conductance, or voltage) during 100–200 ms time windows. To estimate spatial receptive field parameters, we fit Difference-of-Gaussians (voltage, excitation) or single Gaussian (inhibition) models to area response data (Enroth-Cugell and Robson, 1966; McMahon et al., 2004). Receptive field sizes were measured as the diameter of the respective Gaussians at 1 SD. To analyze of VG3-ACs, we recovered separate ON and OFF filters from responses to Gaussian white noise stimulation. First, overlapping stimulus segments were weighted by their ensuing baseline-subtracted response (voltage or conductance) to compute the response-weighted stimulus ensemble, analogous to the spike-triggered stimulus ensemble frequently used in the characterization of RGC responses (Chichilnisky, 2001). We then identified the dimension of highest variance in the response-weighted stimulus ensemble by principle components analysis and separated response-weighted stimulus segments in two groups based on their projection onto this first principle component (positive = ON, negative = OFF). ON and OFF segments were averaged to determine ON and OFF filters, respectively (Figure 1—figure supplement 3). The amplitudes of these linear filters were scaled such that their sum equaled the sum of the global response-weighted stimulus average. Similar procedures have previously been used to analyze the contributions of ON and OFF pathways to RGC spike trains (Fairhall et al., 2006; Greschner et al., 2006; Gollisch and Meister, 2008). To summarize temporal frequency tuning and sensitivity across several cells, Fourier amplitudes of ON and OFF filters were calculated. To characterize spatial integration, Fourier amplitudes of responses at once (F1, 2 Hz) and twice (F2, 4 Hz) the frequencies of sine wave modulation of contrast-reversing square-wave gratings over the receptive field center (excitation) or surround (inhibition) were analyzed. Edge detection tuning was measured from response amplitudes as leading and trailing edges of dark or bright bars of different heights moving at a variety of speeds crossed the receptive field center. Similarly, response amplitudes during selective (center or surround) or global (center and surround) luminance-neutral motion stimuli were compared to reveal differential motion sensitivity.

Synaptic connectivity between VG3-ACs and W3-RGCs was analyzed in confocal image stacks of retinas from *VG3-Cre Ai9* mice in which a sparse subset of RGCs (incl. W3-RGCs) expressed CFP and PSD95-YFP following biolistic transfection. Using local thresholding W3-RGC dendrites, PSD95 puncta and VG3-AC neurites were masked separately in Amira (FEI Company). Excitatory synapses on W3-RGCs formed by VG3-ACs were defined as PSD95 clusters with a center of mass within 0.5 μm from a VG3-AC neurite. We confirmed that varying this distance from 0.25 to 1 μm did not qualitatively change the results. Given the size of synaptic puncta, this range implies overlap or direct apposition of signals from PSD95-YFP and tdTomato in VG3 neurites. To compare the input fraction (i.e., fraction of PSD95 puncta apposed by a VG3 neurite) to chance levels, positions of PSD95 puncta were randomized within the synaptic layer in Monte Carlo simulations. Territories occupied by neurites of VG3-ACs and dendrites of W3-RGCs were measured by the area of the smallest convex polygon to encompass the respective arbors in a z-axis projection of image stacks acquired in flat-mounted retinas. To analyze soma distributions of VG3-ACs, we used a previously described algorithm to automatically identify cell positions (Soto et al., 2012) and calculated the density recovery profiles of these positions (Rodieck, 1991).

Throughout this study, paired and unpaired t-tests were used to assess statistical significance of observed differences.
